# A comparison of DALYs for periodontal disease in China between 1990 and 2013: insights from the 2013 global burden of disease study

**DOI:** 10.1186/s12903-017-0356-7

**Published:** 2017-04-11

**Authors:** Qi Zhang, Zhixin Li, Chunxiao Wang, Yunning Liu, Yang Yang, Scottie Bussell, Maigeng Zhou, Linhong Wang

**Affiliations:** 1 National Center for Chronic and Non--Communicable Disease Control and Prevention, Chinese Center for Disease Control and Prevention, 27 Nanwei Road, Xicheng District, Beijing, 100050 China; 2 Parker Indian Health Center, 12033 West Agency Road, Parker, Arizona 85344 USA

## Abstract

**Background:**

China has undergone a rapid demographic and epidemiological transition with fast ecomonic development since the 1980s. Oral health is becoming a major public health problem as the prevalence of non-communicable diseases has greatly increased. Periodontal disease (PD) and caries are among the most prevalent oral diseases. PD accounts for the majority of tooth loss and increases with age. China’s third national epidemiological investigation on oral diseases (2005) revealed that periodontitis affected >50% of the adult population. The Global Burden of Disease Study 2013 (GBD2013) have been used to estimate DALYs for 301 acute and chronic diseases and injuries in 188 countries for 1990–2013. The estimation of burden of PD between 1990 and 2013 will provide a unique perspective for planning interventions and developing public health policies for PD even chronic diseases in China.

**Methods:**

We used the GBD 2013 results for Years of Life Lost (YLLs) and Years Lived with Disability (YLDs) to calculate Disability Adjusted Life Years (DALYs) for PD in China. PD standardized DALYs rate (SDR) per 100,000 persons, the percentage of PD standardized DALYs rate (% PD SDR) in all diseases DALYs, and variance ratio of these two indexes between the years of 1990 and 2013 were compared by province, gender and age groups.

**Results:**

Nationwide, compared to 1990, the SDR in 2013 increased slightly from 24.7 to 25.7, while the variance ratio of SDR for provinces in the middle, west and south of China showed a greater variation(4.8–6.2%). The % PD SDR in all disease DALYs increased from 0.06 to 0.11% for all groups. The four highest variance ratios % PD SDR in all diseases DALYs between 1990 and 2013 occurred in the west of China (97, 98.6, 108.4 and 112.8%). The PD SDR changed slightly in the women (20.3 to 21.7), meanwhile the variance ratio of PD SDR and % PD SDR in all diseases DALYs for the women (6.7 and 94.5%) was also higher than for men (2.1 and 60.6%). The highest variance ratio % PD SDR in all oral diseases DALYs occurred between 1990 and 2013 in ages 20 to 24 (50.7%) and 25 to 29 years (50.5%).

**Conclusion:**

The PD standardized DALYs rate and % PD SDR in all diseases DALYs in China in 2013 has increased from 1990. Especially, the variance ratio of % PD SDR in all disease DALYs among Young population and women, in the west provinces of China have been becoming the highest in all age groups and national wide. Future intervention measurements should include young women of child-bearing age because women’s health impacts infant health. Periodontal disease has risk factors in common with a number of other non-communicable diseases (NCD) and conditions, and focusing on the common behavioral and environmental risk factors would be instrumental in the effective prevention of periodontal disease.

## Background

According to the WHO, the prevalence of periodontal diseases (PD), which includes gingivitis, gum bleeding, calculus, and periodontitis remains high in developing countries [1]. Perhaps of 10 to 15% of adult are afflicted by PD, with an increasing prevalence with age [2]. PD contributes to diabetes mellitus, cardiovascular disease and other systemic disorders and vice versa [3]. This association likely exists because of common risk factors like tobacco use, poor diet, excessive alcohol consumption, stress, and unhygienic practices such as poor teeth brushing [4]. Chronic PD was shown to adversely affect the quality of life of people with type 2 diabetes living in China by impacting chewing, socialization and speech [5].

China’s third national epidemiological investigation on oral diseases (2005) revealed that periodontitis affected >50% of the adult population. Gingival bleeding and calculus occured in most 12-year-old (57.7, 59.1%), middle-aged adults(77.3, 97.3%) and elderly (68.0, 88.7%). In aging populations, 70–90% of individuals persons 60 and 74 suffered from PD [6]. Drury et al reported there was a 10 to 20% difference in periodontal disease prevalence and severity between people of higher and lower socioeconomic status in the U.S. population [7].

The Global Burden of Disease Study 2013 (GBD2013) have been used to estimate DALYs for 301 acute and chronic diseases and injuries in 188 countries for 1990–2013 [8]. DALYs provide an overall estimate of the health of a population that accounts for years spent in ill health or disability. The general approach for the definition of PD of GBD 2013 was similar to that for GBD 2010. The GBD 2010 definition of severe periodontitis were based on “a Community Periodontal Index score of 4, a clinical attachment loss more than 6 mm or a gingival pocket depth more than 5 mm”, depending on which was used in the publication [9]. Kassebaum et al [10] provided the process including search strategy for identification of studies, selection of studies and data extraction and cleaning, data handling and modeling for estimation of prevalence and incidence for severe periodontitis in GBD 2010. Also, GBD 2010 data have used to estimate the PD burden in Global and Iran [9–11].

The aim of our study is to describe DALYs in China for PD by province, sex, and age groups. To our knowledge, this is first study to estimate the burden of PD in China using GBD2013 data. The estimation of this study will provide a unique perspective for planning interventions and developing public health policies for PD even chronic diseases in China.

## Methods

### Data source

Detailed methods for each component of the GBD 2013 and China estimation are described elsewhere [12, 13]. We provide a brief description here, with emphasis on PD. We used five main data sources that provide information about causes of death. Across systems, data have been reported using two versions of ICD-9 tabulations and one of ICD-10 (panel). These have all been mapped to the GBD cause list [14].

YLLs were computed through using multiply numbers of deaths from each cause in each age group by the reference life expectancy at the average age of death for those who die in the age group following the standard GBD 2010 methods [15]. YLDs were calculated by multiplication of prevalence, the disability weight for a sequel, and the duration of symptoms. Prevalence estimates were assessed using the database for all age-sex-country-year groups, with a Bayesian meta-regression method (DisMod-MR) [9]. We used the GBD 2013 appointed results for YLLs and YLDs to calculate DALYs for PD in China. Specially, since deaths directly attributed to oral disease is a rare event, DALYs estimates were based on YLDs only.

The DALYs in 1990 and 2013 and the variance ratio of DALYs between 1990 and 2013 were stratified by province (*n* = 33), gender, and age. We analyzed data from all 33 provinces in China including Hong Kong and Macao Special Administrative Regions, which we refer to as provinces in this study. The estimation of GBD2013 were stratified ages 14 to ≥ 80 into 14 groups in 5-year intervals for analysis, which was also coincident with the average time from gingivitis and gum bleeding development to periodontitis in adults [16].

### Statistical analysis

The data of DALYs of PD in 1990 and 2013 of China were available from Global Health Data Exchange (GHDx), here described and organized respectively. The quantity of DALYs in this report included standardized DALYs rate (SDR) per 100,000 persons and the percentage of PD DALYs (% PD SDR) in all diseases DALYs. SDR was calculated as: number of mean years divided by the population census in 2010 multiplied by 100,000 persons and Variance ratios of DALYs between 1990 and 2013 were calculated according to the GBD 2013 Diamond-MR 2.0 models. The value of two quantities were shown as mean DALYs and 95% uncertainty intervals (UIs). These UIs included uncertainty that stems from the sample size of data, adjustments to different sources of all-cause mortality, and cause-of-death model specification and estimation. Uncertainty from all of these sources was propagated into the final quantities of interest by taking 1,000 draws from the posterior distribution of each component quantity of interest [13].

ArcInfo GIS software V. 10. 2 (Environmental Systems Research Institute, Redlands, CA, USA) and Microsoft office excel 2007 were used to organize the 1990 and 2013 data into tables and charts and to illustrate the DALYs changes among 33 provinces on the China map.

## Results

### Province-specific DALYs for periodontal disease in 1990 and 2013

**Fig. 1:**
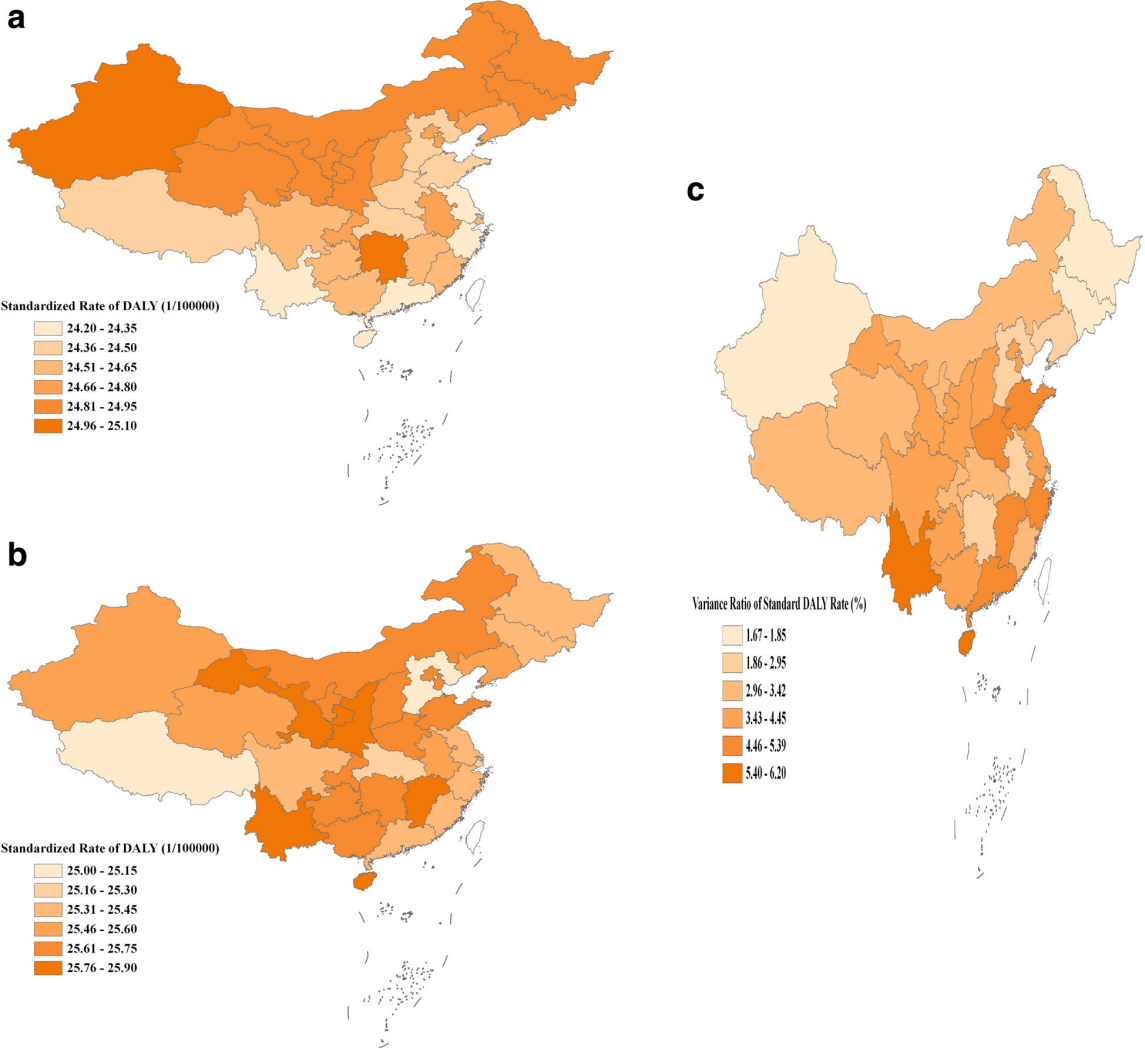
Province level displayed the periodontal disease standardized DALYs rate per 100, 000 persons in 1990 and 2013 in China. **a** PD Standardized DALYs rate per 100,000 persons of 1990. **b** PD Standardized DALYs rate per 100,000 persons of 2013. **c** Variance ratio of PD standardized DALY rate between 1990 and 2013

We estimated the PD SDR per 100, 000 persons for 33 provinces for all ages. In nation-wide and all age group, compared to that of 1990, the SDR in 2013 increased slightly from 24.7 to 25.7. Although the SDR for 33 provinces were close in 1990 and 2013, in 1990 the largest scale of SDR among 33 provinces was 0.9. Guangdong and Zhejiang had the lowest SDR at 24.2. Macao Special Administrative Regions, Hunan and Xinjiang Uyghur Autonomous Region had the highest SDR at 25.1. The SDRs in the southern and eastern region of China were relatively lower than those of north and northeast region (Fig. 1a). The map pattern of SDR in China in 2013, however, showed provinces with relatively higher SDR (≥25.6) were mainly clustered in the region of northwest, north and the southwest of China (Fig. 1b). In 2013, the province with the lowest SDR was Hebei at 25.0; conversely, four provinces including Gansu, Shannxi, Yunnan, and Jiangxi had the highest SDR at 25.9.

Comparing variance ratio of SDR between 1990 and 2013, we can see that provinces in middle, west, and the south of China had a relative large variance from 4.8 to 6.2% between 1990 and 2013 (Fig. 1c). Provinces such as Shandong, Hainan, and Yunnan had the largest variance ratios (5.4–6.2%) compared to northern provinces such as Shanxi, Hebei, Beijing, Tianjin.

**Fig. 2:**
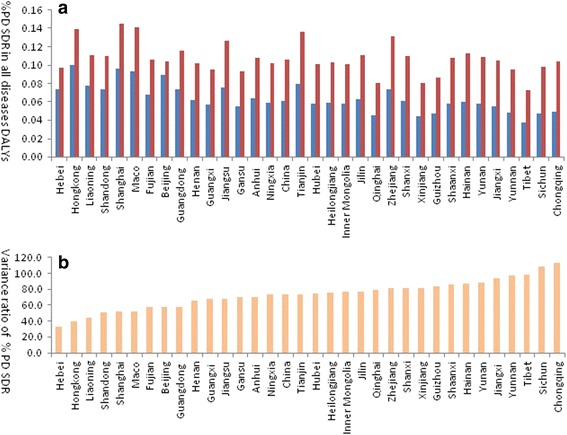
The percentage of PD standardized DALYs rate in all diseases DALYs in 1990 and 2013 as province displaying. *Blue* bar represent data of 1990, *Red* bar represent 2013’s. **a** Provincial % PD SDR in all diseases DALYs in 1990 and 2013. **b** Provincial variance ratio of % PD SDR in all diseases DALYs between 1990 and 2013

Over 23 years, the % PD SDR in all diseases DALYs increased almost two times from 0.06 to 0.11% for all people. DALYs contributed by % PD SDR grew in all 33 provinces from 0.07 to 0.14% in 2013. Eighteen provinces had a higher % PD SDR in all diseases DALYs (0.11 to 0.14%) than that of the rest of China (Fig. 2a). Only six provinces including Hebei, HongKong, Liaoning, Shanghai, Guangdong and Macao had lower % PD SDR in all disease DALYs from 0.07 to 0.09% (Fig. 2a). The four highest variance ratios (96.98, 98.63, 108.45 and 112.77%) % PD SDR in all disease DALYs between 1990 and 2013 occurred in west provinces including Yunnan, Tibet, Sichuan and Chongqing. The other 14 provinces had higher variance ratio of % PD SDR in all diseases DALYs. The 15 provinces that had a lower variance ratio % PD SDR in all diseases DALYs than of Chinese provinces were mainly scattered in the middle and eastern part of China (Fig. 2b).

### Sex specific periodontal DALYs in 1990 and 2013

In contrast to men, the PD SDR per 100,000 persons changed slightly for women from 20.3 to 21.7. However, the variance ratio of PD SDR for women was higher than for men (6.7% vs 2.1%). Although the % PD SDR in all diseases DALYs was equal as 0.11% between man and women in 2013, the variance ratio % PD SDR in all diseases DALYs was noticeably higher for women compared to men (94.5% vs 60.6%) (Table 1).

**Table 1:** Gender groups specific PD standardized DALYs and variance in 1990 and 2013

Gender	Standardized DALYs rate(per 100,000 persons)			Percentage of PD SDR in all diseases DALYs		
	1990	2013	Variance ratio	1990	2013	Variance ratio
Male	29 (11.6–59.6)	29.6 (11.9–60.1)	2.1 (−1.7–6.1)	0.07 (0.03–0.13)	0.11 (0.04–0.21)	60.6 (44.2–79.0)
Female	20.3 (8.1–41.5)	21.7 (8.7–44.3)	6.7 (3.3–10.8)	0.06 (0.02–0.11)	0.11 (0.04–0.21)	94.5 (75.2–115.3)
All	24.7 (9.9–50.6)	25.7 (10.3–52.3)	3.9 (1.2–6.8)	0.06 (0.03–0.12)	0.11 (0.04–0.21)	74.3 (60.5–89.3)

### Periodontal disease DALYs increased with age in 1990 and 2013

**Fig. 3:**
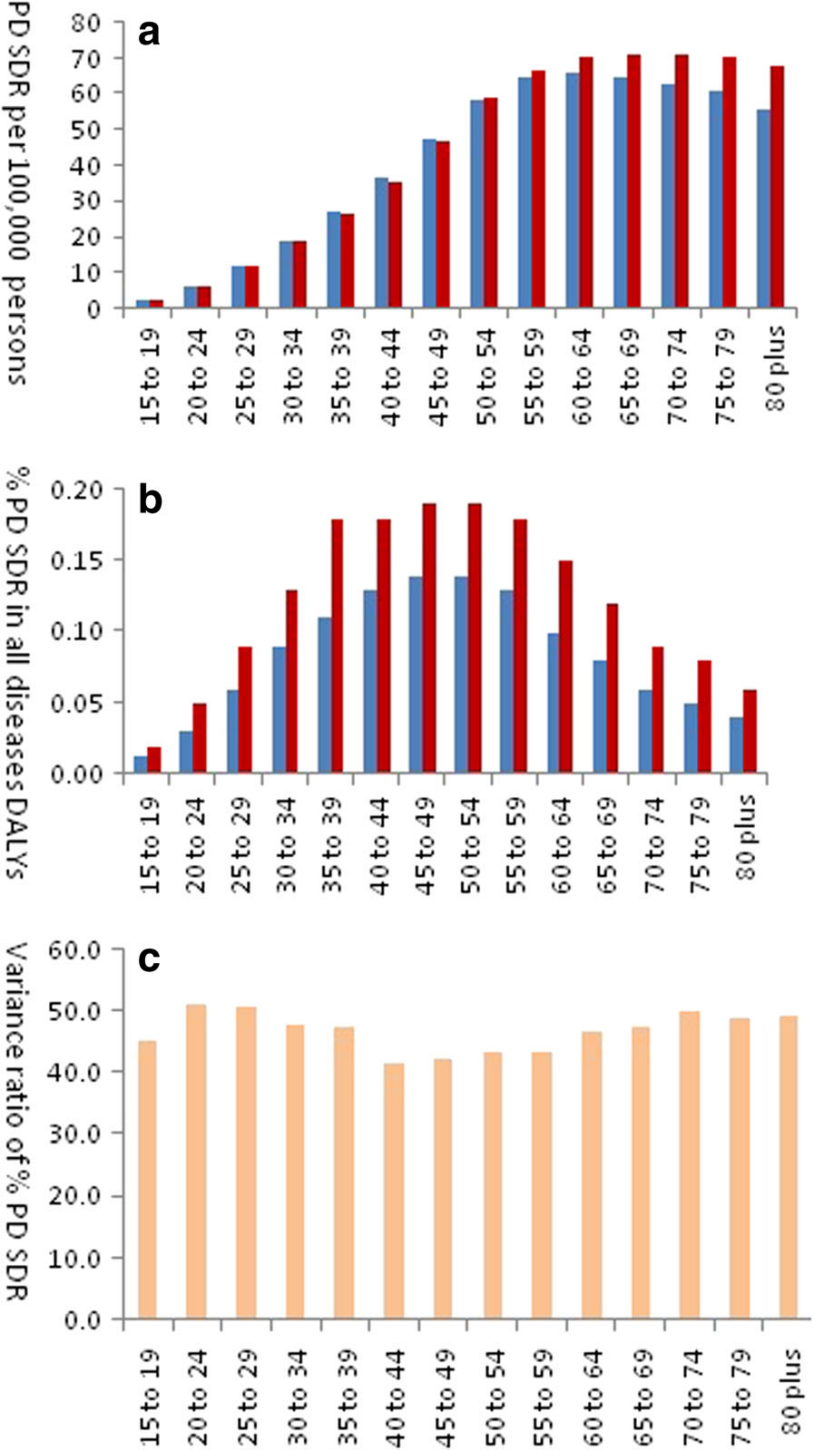
PD standardized DALYs rate of age groups in 1990 and 2013. *Blue* bar represent the data of 1990, *red* bar represent the data of 2013. **a** PD SDR per 100,000 persons of 14 age groups in 1990 and 2013. **b** % PD SDR in all diseases DALYs of 14 age groups in 1990 and 2013. **c** Variance ratio of % PD SDR in all diseases DALYs of 14 age groups between 1990 and 2013

PD SDR among all 14 age groups in 2013 increased slightly more than that in 1990. Moreover, the increase pattern of PD SDR among 14 age groups in 2013 was similar with that of 1990. In 1990, the SDR increased gradually from 1.8 for ages 15 to 19 years to 58.5 for persons 50 to 54 years. There was very little change from the group of 55 to 59 years to the group of 75 to 79 years with 64.8, 66.1, 64.5, 62.6 and 60.5 respectively. As shown in Fig. 3a, in 2013, persons over 60 years of age had the highest SDR around 70.0.

The % PD SDR in all disease DALYs increased from 1990 to 2013 in all age groups. In both 1990 and 2013 there was the highest % PD SDR in all disease DALYs for persons 45–54 year of age compared to other groups. However, the highest variance ratio of % PD SDR in all disease DALYs were in the age groups 20–24 years (50.7%) and 25 to 29 (50.5%) (Fig. 3c).

## Discussion

PD SDR and the % PD SDR in all disease DALYs increased over 23 years. In nation-wide and all age population, compared to that of 1990, the SDR in 2013 increased slightly from 24.7 to 25.7. DALYs contributed by the % PD SDR for China was 0.06% in1990 and 0.11% in 2013. The west part of China had significantly higher variance ratio of PD SDR and % PD SDR in all diseases DALYs between 1990 and 2013 than those of other China parts. China’s DALYs estimation was much lower than that of global and Iran previously reported in 2010. Marcenes et al reported on the global DALYs of severe periodontitis was 97 and 108 in 1990 and 2010, respectively. DALYs in Iran did not noticeable change for periodontal diseases from 68.6 to 67.6 in 1990 and 2010. But the periodontal disease contributed to 0.8% of the total DALYs in 1990 and 2010 in Iran, which is less change than that of China.

The special outline of DALYs estimation in China have its specific explanation with the transition pattern of diseases occurrence among Chinese people during the period of more than 30 years. Firstly, China has undergone a rapid demographic and epidemiological transition during these times. Oral health is becoming a major public health problem as the prevalence of non-communicable diseases has greatly increased. According to a 2010 surveillance report, the estimated prevalence of diabetes among Chinese adults was 11.6% [17]. During these 30 years, China has been conducted four national oral health epidemiology investigations. The 1995 and 2005 surveys indicated that the oral health status of the Chinese population has not improved with an increasing number of dentists and oral health institutions [18]. Meanwhile, the number of retained teeth among urban adults aged 65–74 years increased from 18.80 to 21.98. These conditions resulted in a increased burden of periodontal diseases.

Secondly, China’s tobacco consumption increased at an annual rate of 5.3% starting in 1996. This increase could explain the parallel increased prevalence of PD and its related PD SDR over the last 23 years. Finally, more Chinese are being diagnosed with PD in selected areas. An investigation of national oral health departments in the Center for Disease Control and Prevention system was conducted in more than 3,000 provinces, city and county CDC institutes in 2013 (unpublished data). The results showed that 18 provincial CDCs, located in middle and western China, were cooperating with local oral technical institutes such as dental hospitals. Thus, these areas had local oral health promotion programs including free oral examination, oral health behavior guidance and oral health knowledge education. These areas had greater awareness of oral health and greater access to care. More patients were diagnosed with oral disease as well. Thus, the higher variance ratio of PD SDR and % PD SDR in all diseases DALYs observed in this study in the west areas of China may be explained by the differences in knowledge and coverage of oral care.

From China’s estimation, in contrast to men, the PD SDR changed slightly for women from 20.3 to 21.7. The variance ratio % PD SDR in all diseases DALYs was noticeably higher for women compared to men (94.5% vs 60.6%). Based on the China Adult Tobacco Investigation Report (2015), 52.1% of men and 2.7% of women smoke [19]. Studies showed that the PD standardized DALYs rate of GBD 2013 in China was worse for men compared to women [20, 21]. Some investigators believed that the burden of PD was higher in China for men because more men smoke compared to women and that smoking was a risk factor for tooth loss [22, 23]. However, the variance ratio of PD standardized DALYs rate and % PD SDR in all oral diseases DALYs in women were higher than that of men, which may reflect health and education disparities in women.

PD SDR among all 14 age groups in 2013 increased slightly more than that in 1990. The highest variance ratio of % PD SDR in all disease DALYs were in the age groups 20–29 years (50.5%), Compared to middle and older age adults, young Chinese adults had a lower social and economic status. We can inferred that 1) young adults with low income have financial barriers to accessing oral health care; 2) are less likely to be aware of the need for comprehensive, ongoing dental care, and 3) are more likely to use tobacco and have a poor lifestyle [24]. Further, young adults with lower education may lack adequate oral health knowledge, not engage in preventive behaviors, and may under use oral health services [25]. Sabbahet al argued that access to dental care is the main factor in socioeconomic disparities in oral health [26]. So, much more young adults were diagnosed with PD.

This study has several limitations. First, our conclusions are limited by the quality of data inherent in GBD 2013 such as sampling and non-sampling error, model specification, and model parameter estimation [27]. Further, describing DALYs for PD is less straight forward compared to other chronic diseases such as mental illness. Disability may be subtler, such as decline in nutrition over many years or overshadowed by a person’s other co-morbidities. Thus, DALYs may only be one of many tools to quantity a nation’s oral health. The incidence of PD should be integrated with the DALYs to accurately estimate the disease burden of PD. The fourth national oral health epidemiological investigation began at the end of 2015 and will conclude in 2017. The results will help to better determine the true burden of oral health diseases.

## Conclusion

We conclude that the PD standardized DALYs rate in China in 2013 has increased from 1990. The % PD SDR in all diseases DALYs also increased over 23 years. Especially, the variance ratio of % PD SDR in all disease DALYs among Young population and women, in the west provines of China have been becoming the highest in all age groups and national wide. Periodontal disease has risk factors in common with a number of other non-communicable diseases (NCD) and conditions, and focusing on the common behavioral and environmental risk factors would be instrumental in the effective prevention of periodontal disease. More attention should be placed on younger populations and women who will contribute the most DALYs. Future intervention measurements should include young women of child-bearing age because women’s health impacts infant health [28].

## Abbreviations

% PD SDR: Percentage of PD standardized DALYs rate
DALYs: Disability adjusted life years
GBD 2013: Global burden of disease study 2013
PD: Periodontal disease
SDR: Standardized DALYs rate
YLDs: Years lived with disability
YLLs: Years of life lost
